# Things Become Appealing When I Win: Neural Evidence of the Influence of Competition Outcomes on Brand Preference

**DOI:** 10.3389/fnins.2018.00779

**Published:** 2018-10-26

**Authors:** Wenjun Yu, Zhongqiang Sun, Taiwei Xu, Qingguo Ma

**Affiliations:** ^1^Business School, Ningbo University, Ningbo, China; ^2^Academy of Neuroeconomics and Neuromanagement, Ningbo University, Ningbo, China; ^3^Department of Psychology, Ningbo University, Ningbo, China; ^4^Institute of Neuromanagement Science, Zhejiang University of Technology, Hangzhou, China

**Keywords:** brand preference, victory and defeat, emotion, event-related potentials, neuromarketing, neuromanagement

## Abstract

Against the background of an increasingly competitive market environment, the current study aimed to investigate whether and how victory and defeat, as two critical factors in competition outcomes, would affect consumers’ preference of unfamiliar brands. In the experiment, participants’ status of victory or defeat was induced by a pseudo-online game, followed by a main task of brand preference rating. Using the precise and intuitive attributes of neuroscientific techniques, we adopted event-related potentials to analyze brain activity precisely during brand information processing when individuals experienced victory or defeat. Behavioral data showed that individuals had a stronger preference for unfamiliar brands in victory trials than in defeat trials, even if the brand was completely unrelated to the competition; this indicated a transfer of valence. Three emotion-related event-related potential components, N1, P2 and later positive potentials, were elicited more negatively in victory trials than in defeat trials, indicating the existence of incidental emotions induced by victory or defeat. No significant correlation was found between any pair of ERP components and preference scores. These results suggest that the experience of victory and defeat can evoke corresponding incidental emotions without awareness, and further affect the individual’s preference for unfamiliar brands. Therefore, playing a game before presenting brand information might help promote the brand by inducing a good impression of the brand in consumers.

## Introduction

In modern society, competition between two or more organisms is ubiquitously present, ranging from economic competition and an arms race between countries, to rivalries among colleagues and schoolmates. Cruelly, victory and defeat always accompany competition, which may have an impact on an individual’s attention (Sun et al., 2015), perception (Yu et al., 2017), emotion (Aviezer et al., 2012, 2015), confidence (Hsu and Wolf, 2001), and even sense of control (Burger, 1989). This would further result in numerous subsequent behavior changes, such as affecting the individuals’ enthusiasm to participate in a contest (Rutte et al., 2006).

Considering the broad cognitive and behavioral impact from victory and defeat, consumer behavior studies have begun to pay attention to the influence of competition outcomes on consumers’ mental status and decision-making process (Gronmo, 1988; Dalton, 2008; Sivanathan and Pettit, 2010). For instance, when participants were given false feedback on tests of their intelligence, those with a low IQ test result increased their willingness for self-expression consumer behavior, such as selecting expensive items, products, and shops (Dalton, 2008). Sivanathan and Pettit (2010) also found that negative test feedbacks induced consumers to bid higher for limited edition photographs. Therefore, consumers’ experience of failure strengthened compensatory consumption, based on their need for improving self-esteem.

However, with the developing global integration of technology and the increasingly fierce market competition, it is becoming more difficult to gain predominance solely based on product differences because products have become more homogeneous. In this setting, brand awareness, which plays a key role in consumer behavior via information involving symbolic and systematized representations, memory, judgment, and inference (Loken, 2006; Jin et al., 2015), is becoming a critical factor affecting consumer behavior. Once the preference for a brand is determined, it tends to endure, be unaffected, and more importantly, is not easily replaced. As one of the main metrics for brand awareness, brand preference has been proven to predict consumer’s brand evaluation, brand choice, as well as purchase intention and behavior (e.g., Priester et al., 2004; Fazio and Petty, 2007). Thus, whether competition outcome would affect brand attitude is an important topic; however, few studies have addressed this issue. Moreover, the psychological process of developing consumers’ “favor” and “disfavor” should receive attention, so that brand preference can be strengthened.

Accordingly, the current study aimed to investigate the influence of competition outcome on brand preference and determined the process of its formation. Therefore, the current study first induced a victory or defeat experience by means of a competition, followed by the major task of brand preference rating. Questionnaires and behavioral experiments are two primary measurements used in research concerning brand preference. These subjective self-report instruments solely rely on participants’ thoughts and description, yet many factors affecting human behaviors cannot be assessed by human conscious awareness (Zaltman, 2002). Furthermore, surroundings and social desirability would also distract from a preference-rating task, resulting in an expectation effect and manifesting as bias between the obtained results and the real mental process (Camerer et al., 2005). To address this issue, neuroscientific methods, such as event-related potentials (ERPs), may be a way to obtain unbiased and objective results. Therefore, we adopted ERP to determine the fundamental patterns of brain activity during brand information processing, while an individual is experiencing victory and defeat, more precisely. In this case, we could infer the neural dynamics and further explore factors that potentially mediate the victory/defeat experience as well as the consumer’s attitude toward unfamiliar brands.

The ABC model of attitudes (Breckler, 1984) suggests that emotion might be one of the influential factors. According to this model, human attitude structure is generally divided into three main component: consumers need to know the commodity first (cognitive), which generates the corresponding emotion (affect), and then make a final consumption decision (behavior) (Breckler, 1984). The indispensable role of emotion in this model has also been supported by empirical research. For instance, Harlé and Sanfey (2007) found that subtle emotions, such as sadness and amusement, induced by short movie clips, could effectively bias the decision-making process in the Ultimate Game. Moreover, distinguishable incidental emotions, which are even unrelated to the immediate situation, would also lead to different product evaluations (Kim et al., 2009). In analogy, in the current study, we inferred that consumer behavior might, to a large extent, be affected by emotional arousal when facing an unfamiliar brand. To reveal the subtle emotion distinction, the ERPs N1 and P2, during the early processing phase, and late positive potentials (LPPs), in the later cognitive phase, which could be evoked at different amplitudes by positive and negative emotional stimuli, were adopted as the ERP indicators in the current study.

During information processing, N1 has been considered as the earliest component induced by emotional information; the amplitudes of this ERP differs notably for positive and negative emotional stimuli (Carretié et al., 2003). N1 is evident at frontal–central sites at approximately 130–150 ms after the onset of an emotion stimulus (Keil et al., 2001). P2 is also an early component that peaks positively at 250–350 ms after the onset of a stimulus, and has been claimed to be a sensitive index of attention distribution, with a higher P2 amplitude elicited by negative stimuli than with positive or neutral stimuli (Carretié et al., 2001; Huang and Luo, 2006; Wang et al., 2012). In the later processing phase, LPP, which is associated with evaluative categorization, generally begins around 300–500 ms after the onset of a stimulus and lasts for hundreds of milliseconds (Cuthbert et al., 2000; Schupp et al., 2000; Wang et al., 2016); it is elicited with greater amplitudes by negative rather than positive stimuli (Hajcak and Olvet, 2008; Ma et al., 2017). We therefore utilized N1, P2, and LPP to examine the role of emotions underlying the whole process from competition outcomes to brand preference.

In the current study, participants were asked to complete a time-estimation task as a means of generating a victory or defeat experience, and then their degree of preference for an unknown brand was assessed. Both behavioral and ERP data were collected to investigate whether victory or defeat experience would affect the individuals’ preference for strange brands. Behaviorally, we assumed that individuals who had experienced victory would demonstrate a stronger preference for an unrelated and unknown brand than individuals who had experienced defeat. Neurologically, the victory experience was expected to induce a more positive emotion, thus a more negative N1, P2, and LPP might be evoked by a victory than by a defeat experience. In addition, the correlation between brand preference rating and brain activities of emotion induced by victory or defeat experience was also considered. If a correlation was evident, brand preference would change linearly with emotion intensity; alternatively, the increase in the degree of emotion intensity would not accompany a widened bias for brand preference.

## Materials and Methods

### Participants

Twenty-one graduate and undergraduate students (11 females; mean age 23 years) were paid to participate in this experiment. None had a history of neurological problems and all had normal or corrected-to-normal vision. The participants provided written informed consent in accordance with the Declaration of Helsinki prior to the experiment, and all experimental procedures were approved by the local institutional ethics committee of the Academy of Neuroeconomics and Neuromanagement at Ningbo University.

The sample size of the study was determined via a “distribution-based approach to test selection” in G^∗^Power 3 (Faul et al., 2007; Nosek et al., 2009; Minichilli et al., 2010). Given a large effect size (*f* = 0.40; $\eta_{p}^{2}$ = 0.14), a power of 0.95, and an alpha level of 0.05, we found that a paired sample *t*-test would be more powerful than a within-subjects *F*-test. Thus, we used the within-subject *F*-test for consideration of power. The power analysis ultimately yielded an estimated sample size of 18. Furthermore, considering drop-out and exclusion, we decided to enroll 24 individuals. Eventually, the data of three participants were discarded due to excessive recording artifacts, resulting in a valid sample size of 21 for analysis.

### Stimuli

**FIGURE 1:**
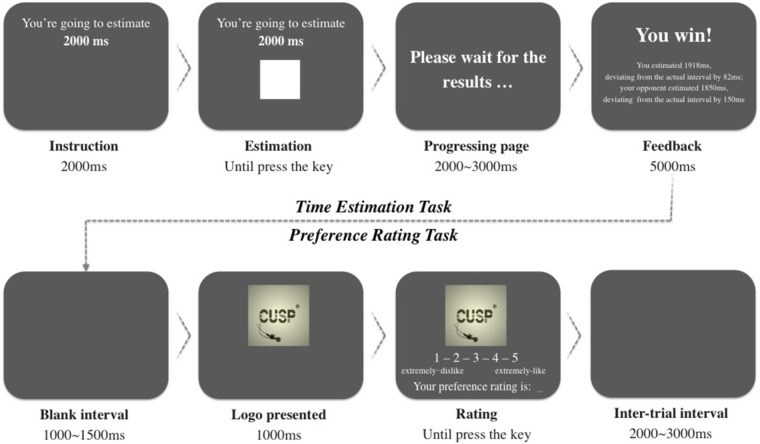
An example of a trial with 2,000-ms estimation time in a victory condition, proceeding from left to right and top to bottom.

Stimuli were presented on a gray background (80, 80, 80) monitor of a 19-inch computer (100-Hz refresh rate). Two types of stimuli were adopted in the experiment. In the time estimation task, we used a 3.0° × 3.0° white square, always positioned in the center of the screen. In the brand preference rating task, all 40 logo images from the study by Ma et al. (2017) were used. Each logo image comprised English letters representing the brand name and an earphone picture (see Figure 1). The earphone picture was the same across the 40 logos, occupied a 7.0° × 7.0° square area, and were also positioned in the center of the screen. These brand logos do not exist in real life, and no participant in either the study by Ma et al. (2017) or the present study reported having ever seen any of them. In order to minimize the influence of luminance, contrast, and color saturation difference, all logo images were gray-processed using Photoshop^®^.

### Design and Procedure

When the participant entered the laboratory, he/she was told to compete with another participant as the opponent in a LAN-based game. Their communication was restricted to a greeting when they first met at our laboratory. In the formal experiment, the “LAN-based game” was actually an offline game, and the opponent was one of the experimenters, in disguise, who would not in fact play the game. These manipulations were used in order to control the participant’s winning percentage to guarantee sufficient valid trials for ERP analysis.

Participants were seated in an electrically shielded and sound-attenuated recording chamber at a distance of 70 cm from the monitor. We used Presentation^®^ software to control the stimulus presentation and response acquisition. Participants were given clear instructions on how to perform the experimental trials. The procedure is illustrated in Figure 1. Two tasks, the time-estimation task and logo preference-rating task, were set in sequence in each trial. First, the time-estimation task paradigm required participants to estimate an interval as accurately as possible. At the beginning of each trial, an instruction was presented for 2,000 ms to indicate the interval that needed to be estimated, followed by a white square indicating the onset of the time-estimation task. Participants were asked to press a button with their right index finger once they thought that the interval had elapsed. The square would then disappear, and be replaced by a progressing page to wait for the competition results, which lasted 2,000–3,000 ms. Feedback was then given visually, informing the participants whether they had won or lost, the estimated times, and the precision of both their own and their opponent’s time estimations. As an example of a victory condition, the feedback information might be as follows: “You win! You estimated 1,918 ms, deviating from the actual interval by 82 ms; your opponent estimated 1,850 ms, deviating from the actual interval by 150 ms.” In this frame, the participant’s actual estimated time was given, while the opponent’s estimated time was conditionally controlled by the program. In the victory condition, the opponent’s absolute value of the estimated time deviation was larger than that of the participant (randomly generated from 50 to 400 ms); in the defeat condition, the opponent’s absolute value of the estimated time deviation was smaller than that of the participant (randomly generated from 1 to 50 ms). The feedback information was presented for 5 s. After a blank interval of 1,000–1,500 ms, a brand logo image was displayed, indicating the onset of the second task. After a 1,000-ms display, a 5-point Likert scale was presented below the image, and participants were asked to rate their preference for this brand by pressing the corresponding numeric keys on the keyboard, from 1 (extreme dislike) to 5 (extreme like). The rating score would be shown in real time for participants to confirm by pressing the return key. No time limitation was set for the logo-rating task. The inter-trial interval was randomized from 2,000 to 3,000 ms. After all trials had finished, participants were asked if they were aware of the experimental objective and whether their emotion was aroused during the experiment. No one answered affirmatively to these questions, and the data of these participants were then used for analysis.

Each participant completed 80 trials in total, with 40 trials each in victory and defeat conditions. Forty logo images were randomly ordered; each appeared twice, separated by at least 20 trials. The experiment was divided into six blocks with 5-min breaks between them. Before the formal experiment, participants were given the opportunity to practice with other brand logos for at least 15 trials to ensure that they understood the instructions.

### Electrophysiological Recording and Analyses

EEG recordings were made at 64 scalp sites by using Ag/AgCl electrodes mounted on an elastic cap. All recordings were made using the left mastoid reference, and the data were re-referenced offline to the algebraic average of the left and right mastoid voltages. Vertical electro-oculograms (EOGs) and horizontal EOGs were recorded using two pairs of electrodes. One pair was placed above and below the left eye, and the other pair was placed at the outer canthus of both eyes. All inter-electrode impedances were maintained below 5 kΩ. The EEG and EOG signals were amplified by a SynAmps2 amplifier (Compumedics NeuroScan, Charlotte, NC, United States) using a 0.05- to 100-Hz band-pass filter, and were continuously sampled at 500 Hz/channel for offline analysis.

EEG data were analyzed using NeuroScan 4.3.1. The data were initially corrected for eye blinks by using a regression procedure, followed by digital filtering through a zero-phase shift (low pass at 30 Hz, 24 dB/octave). The EEGs were then segmented into epochs ranging from 200 ms before to 1,000 ms after the onset of the logo image for all conditions, and the epoch was baseline-corrected using a 200-ms interval prior to the presentation of the logo image. Trials with remaining artifacts exceeding ±75 μV in amplitude were rejected and excluded from analysis.

Paired sample *t*-tests were conducted on logo preference ratings between the victory and defeat conditions. Two-way repeated-measure analyses of variances (ANOVAs) were adopted to analyze the peak amplitudes of logo-onset N1 and P2, as well as the mean amplitude of LPP during the time-window of interest. Electrode sites in the midline frontal (F1, Fz, F2) and frontal-central (FC1, FCz, FC2) regions were selected for N1 and P2 analysis. To observe LPP, three electrode sites in the parietal region (P1, Pz, P2) were selected, and the amplitudes for the period 300–550 ms were averaged. For factors with more than two levels, the Greenhouse-Geisser correction (Epsilon) was used to adjust the degrees of freedom when necessary. Significant main effects (*p* < 0.05) were always followed by *post hoc* evaluations with a Bonferroni-corrected *p*-value. Pearson’s correlation analyses were also used to assess the correlation between brand preference rating and brain activities of emotion induced by an experience of victory or defeat.

## Results

Behaviorally, the subjective logo preference rating was higher in the victory condition than in the defeat condition, *M_*victory*_* = 3.06, *M_*defeat*_* = 2.89, *t*(20) = −2.48, *p* = 0.022.

**FIGURE 2:**
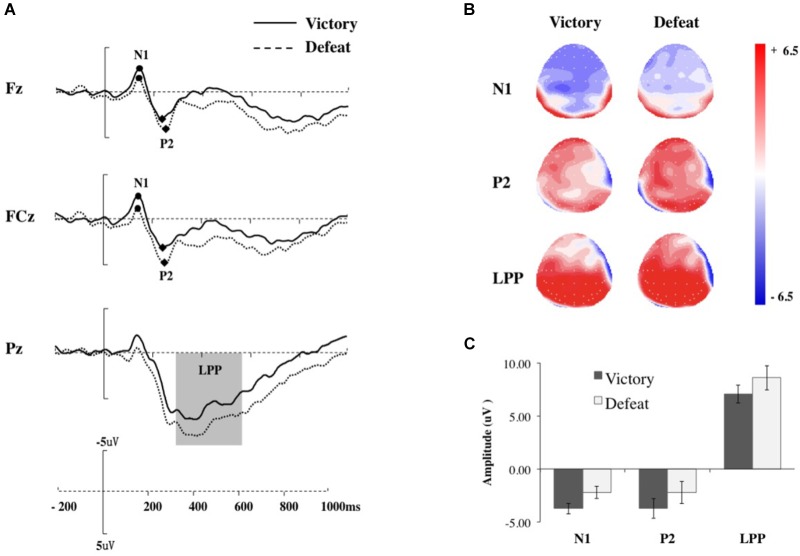
Results of experiments. **(A)** Representative example: ERP waveform in Fz, FCz, and Pz (from top to bottom); **(B)** topographic maps in Victory and Defeat conditions for N1, P2, and late positive potential (LPP); and **(C)** the mean amplitudes of N1 in F1/F2/Fz/FC1/FC2/FCz, and P2 in F1/F2/Fz/FC1/FC2/FCz, and LPP in P1/P2/Pz, and the error bars represent one SEM.

The ERP results are depicted in Figures 2A–C. At brain level, 6 (electrodes: F1, Fz, F2, FC1, FCz, FC2) × 2 (game results: victory, defeat) two-way ANOVA for peak amplitudes of N1 revealed a significant main effect of game results, *F*(1,20) = 6.409, *p* = 0.020, $\eta_{p}^{2}$ = 0.243, while the main effect of electrodes, *F*(5,100) = 0.537, *p* = 0.603, $\eta_{p}^{2}$ = 0.026, and interaction between the two variables were non-significant, *F*(5,100) = 0.859, *p* = 0.436, $\eta_{p}^{2}$ = 0.041. *Post hoc* evaluations confirmed that the averaged peak amplitudes of N1 for the victory condition, *M_victory_* = −3.743, were more negative than those for the defeat condition, *M_*defeat*_* = −2.226.

The peak amplitudes of P2 followed a similar results pattern as N1. A 6 (electrodes: F1, Fz, F2, FC1, FCz, FC2) × 2 (game results: victory, defeat) two-way ANOVA for peak amplitudes of P2 revealed a significant main effect of game results, *F*(1,20) = 4.879, *p* = 0.039, $\eta_{p}^{2}$ = 0.196, while the main effect of electrodes, *F*(5,100) = 1.781, *p* = 0.190, $\eta_{p}^{2}$ = 0.082, and interaction between the two variables were non-significant, *F*(5,100) = 0.918, *p* = 0.414, $\eta_{p}^{2}$ = 0.044. *Post hoc* evaluations also confirmed that the averaged peak amplitudes of P2 for the victory condition, *M_*victory*_* = 4.363, were more negative than those for the defeat condition, *M_*defeat*_* = 5.973.

For LPP, 3 (electrodes: P1, Pz, P2) × 2 (game results: victory, defeat) two-way ANOVA results revealed a significant main effect of game results, *F*(1,20) = 6.133, *p* = 0.022, $\eta_{p}^{2}$ = 0.235. *Post hoc* comparisons showed a markedly more positive amplitude for the defeat condition than for the victory condition. Neither the main effect for electrodes, *F*(2,40) = 1.642, *p* = 0.206, $\eta_{p}^{2}$ = 0.076, nor the interaction between the game results and electrodes, *F*(2,40) = 3.055, *p* = 0.058, $\eta_{p}^{2}$ = 0.132, was statistically significant.

In addition, none of the correlations between preference rating and the mean amplitude of the selected electrodes or either N1, P2, and LPP in Victory and Defeat conditions were significant (*p*s > 0.48).

## Discussion

In the current study, a time-estimation task was adopted to prime victory or defeat, followed by a measurement of the individual’s preference for unfamiliar brands. In this way, we investigated whether the experience of victory and defeat had an impact on brand preference, and assessed the evidence at brain activity level. Behavioral results showed that participants would have a relatively higher preference score for unfamiliar brands after victory trials than after defeat trials. At brain level, more negative N1, P2, and LPP amplitudes were elicited in victory trials than in defeat trials, indicating that individuals’ brand preference was influenced by an incidental emotion induced by the experience of victory or defeat. Moreover, no significant correlation was found between any pair of ERP components and preference ratings.

Consistent with our hypothesis, behavioral data reflected that individuals had a relatively strong preference for unfamiliar brands after experiencing victory, even though the brand information had nothing to do with the competition itself. It could be inferred that the competition outcomes modulated the processing of brand information, manifesting as a transfer of valence.

Furthermore, the results for all investigated ERP components were compatible. A victory experience elicited a more negative N1 in the frontal and central electrode sites than a defeat experience. Previous findings had showed a relatively more negative N1 for emotional stimuli than for non-emotional stimuli, while the amplitudes of N1 for stimuli with positive and negative valences differed markedly (e.g., Carretié et al., 2003). Thus, it is possible that victory and defeat experiences would arouse opposite emotions, and then further induce different impacts on the preference for brands during the early processing phase.

For the sequential P2 component, a larger amplitude was found during the brand preference task after a defeat experience than after a victory experience. As a manifestation of negativity bias, P2 with larger amplitude reflects a greater attention distribution to negative stimuli (e.g., Carretié et al., 2001). Given this cognitive function of P2, the effects of significantly higher P2 amplitude after victory trials in the present study might be due to an induced positive emotion after a victory experience, which is in line with the inference for N1.

For LPP in the later processing phase, the higher central-parietal LPP amplitude found in this study was similar to that in many previous studies on LPP. For instance, a face showing a negative emotion would elicit a larger LPP than a face showing a positive emotion (Ma et al., 2017), demonstrating that faces with different emotional valence reflect differently in terms of LPP. Another study also found a numerically larger LPP for unpleasant than for pleasant pictures (Hajcak and Olvet, 2008). In our study, a similar LPP pattern implied that victory and defeat experiences might undergo an analogical implicit emotion regulation. Taken together, our results were highly consistent across three ERP components: experiences of victory and defeat elicited positive and negative emotions, respectively.

Combining the results of N1, P2, and LPP, we inferred that the initial perception of a new brand undergoes the following stages. A positive or negative emotion is first evoked by an experience of victory or defeat; then, attentional distribution is biased for the subsequent stimuli (even stimuli that are completely unrelated to the competition) during the maintenance period and finally affects the decision-making process. Specifically, in the current study, compared to the Defeat experience, the Victory experience would attract more attentional resources to process the sequentially emerging, irrelevant information, thus increasing its favorability and resulting in behaviorally higher preference scores. Considering the non-significant correlations between each pair of ERPs and behavioral data, we further inferred that such a preference bias only existed between different emotional valences, unrelated to the emotional intensity. Thus, the preference bias occurs when emotional arousal exceeds a certain threshold and is then maintained steadily, regardless of increased emotional intensity.

In line with the ABC model of attitudes (Breckler, 1984), both behavioral and ERP results suggest that competition outcomes would evoke corresponding emotions, which can be considered as incidental effects (Bodenhausen, 1993; Cohen et al., 2008), and then further influence the subsequent brand preference evaluation. Past research has indicated that incidental affect might be involved in various cognitive processes, including consumers’ evaluation of products (Kim et al., 2009), decision-making (Harlé and Sanfey, 2007), and evaluation of brand extensions (Barone et al., 2000; Yeung and Wyer, 2005), mainly demonstrating an affect-congruent influence. Specifically, a good mood always accompanies positive appraisal, while a bad mood accompanies negative appraisal. For example, individuals being interviewed on a sunny day would mostly feel happy and report higher levels of life satisfaction than those being interviewed on a rainy day (Schwarz and Clore, 1983). Thus, positive emotion and behavior become connected due to their close temporal or spatial relationship, as will negative emotion and behavior. According to the “affect-as-information” hypothesis (Schwarz and Clore, 1983; Schwarz, 2012), both decision-related affect and decision-unrelated incidental affect have informational value that increases the cognitive availability of affect-congruent information, and impact the individual’s judgment.

The ERP data from this study have proven that victory and defeat induce different incidental emotions, and bias the preference for the sequentially displayed brand, resulting in an assimilation effect. We considered this process under the “affect-as-information” hypothesis. Since human are not purely rational, we heuristically generate a feeling about an object. In the present study, in which all brands were completely unfamiliar, a sensible evaluation might be influenced only by the current emotional state, and the mood induced by the outcome of the immediately prior competition is the most likely source of this influence. Moreover, the transfer procedure from the incidental emotion to the target object was very rapid, because of the short interval between the time-estimation task and the brand preference-rating task, and may not have been realized by participants, as no awareness of emotional arousal was subjectively reported.

Thus, given the unconsciousness nature and rapid transfer of incidental affect, competition may be utilized as a potent tool to focus consumers’ attention, lift their mood and gain positive brand attitude, without their awareness of the emotion’s effect. In terms of marketing, competitions could accompany the brand of a commodity. In practical use, presenting information about a commodity or brand soon after winning in a pre-set game might help promote the commodity, by leaving consumers with a good impression of this commodity or brand. Furthermore, to benefit from the popular online consumption environment, commodity information can be presented along with individual or interactive online, rapid, and low-cost games as a new marketing approach, so that a positive emotion can be effectively evoked to obtain approval for the subsequently presented commodity, and increasing consumption potential. In an advertising case, such as a lottery, once the winnings are fixed, it would be better to choose that more individuals win a lower prize, rather than having the winnings divided among fewer people. In this way, more interest would be raised from potential clients, which would increase the overall preference for the brand.

## Author Contributions

WY and QM conceived and designed the experiments. WY, ZS, and TX performed the experiments and analyzed the data. WY and ZS compose the manuscript. QM is the principal investigator of the work. All authors reviewed and approved the manuscript.

## Conflict of Interest Statement

The authors declare that the research was conducted in the absence of any commercial or financial relationships that could be construed as a potential conflict of interest.
